# The Plaster-Paris Jacket

**Published:** 1877-09

**Authors:** 


The Plaster-Paris Jacket appears to take very well, not only with the profession, but the public. Its adjustmənt before an audience is so easily made, and the demonstration of its usefulness so impressive, that it has found great favor with many surgeons who are lecturers and teachers. The outside public also appreciate such exhibitions. We wonder how Prof. Sayre will feel to read in a Buffalo newspaper that a surgeon of that place invited a reporter to be present at such an exhibition, and also to read a description of the operation in the said papers. But why not educate the public in these matters, and confine the treatment of such cases to hospital surgeons rather than to ordinary quacks.—*Ibid.*

